# Deagglomeration of Ultrafine Hydrophilic Nanopowder Using Low-Frequency Pulsed Fluidization

**DOI:** 10.3390/nano10020388

**Published:** 2020-02-23

**Authors:** Ebrahim H. Al-Ghurabi, Mohammed Shahabuddin, Nadavala Siva Kumar, Mohammad Asif

**Affiliations:** 1 Department of Chemical Engineering, King Saud University, P.O. Box 800, Riyadh 11421, Saudi Arabia; alghurabi@windowslive.com (E.H.A.-G.);; 2 Department of Physics and Astronomy, King Saud University, P.O. Box 2455, Riyadh 11451, Saudi Arabia; mshahab@ksu.edu.sa

**Keywords:** fluidization, nanopowder, flow pulsation, hydrodynamics, assisted-fluidization, deagglomeration

## Abstract

Low-frequency flow pulsations were utilized to improve the hydrodynamics of the fluidized bed of hydrophilic ultrafine nanosilica powder with strong agglomeration behavior. A gradual fluidization of unassisted fluidized bed through stepwise velocity change was carried out over a wide range of velocities followed by a gradual defluidization process. Bed dynamics in different regions of the fluidized bed were carefully monitored using fast and sensitive pressure transducers. Next, 0.05-Hz square-wave flow pulsation was introduced, and the fluidization behavior of the pulsed fluidized bed was rigorously characterized to delineate its effect on the bed hydrodynamics by comparing it with one of the unassisted fluidized bed. Flow pulsations caused a substantial decrease in minimum fluidization velocity and effective agglomerate diameter. The frequencies and amplitudes of various events in different fluidized bed regions were determined by performing frequency domain analysis on real-time bed transient data. The pulsations and their effects promoted deagglomeration and improved homogeneity of the pulsed fluidized bed.

## 1. Introduction

Ultrafine nanopowders have attracted considerable attention from various scientific and technological fields owing to their unique properties, which mainly arise from their nanoscale dimensions and extremely high specific surface areas. Their uses range from laboratory studies to large-scale applications in catalysis, drug manufacturing, and multipurpose functional material development [1,2,3].

However, effective utilization of nanoparticles in large-scale commercial processes is challenging in view of the agglomeration phenomenon, which tends to shift the particle distribution towards larger sizes. For example, a 12-nm fumed nanosilica sample was found to possess a wide distribution of size that varied from 2 µm to 200 µm [4]. The dimension of a typical agglomerate of nanoparticles (nanoagglomerate) is therefore often more than 1000-fold the actual primary dimension of a nanoparticle. Given that the surface area of any spherical particle varies inversely with the square of its diameter, the formation of an agglomerate that is 1000-fold the size of the particle would inevitably entail 10^6^-fold loss of external surface area of the powder. Therefore, the efficacy of surface area-based rate processes will invariably be compromised because of agglomeration. Moreover, agglomerate formation leads to poor interphase mixing and other bed nonhomogeneities, thereby severely affecting the effective utilization of nanopowder in actual practical applications.

Apart from having high heat and mass transfer rates and intimate phase contact, fluidized beds also improve process energy efficiency by limiting pressure drop to effective bed weight unlike the fixed bed modes of gas-solid contact systems, in which frictional pressure loss generally varies inversely with the square of a particle’s diameter. The fluidized bed mode of contacting can therefore employ small particles as catalytic supports and thereby enhance the effectiveness factor and ensure efficient catalyst utilization. However, a proper understanding of fluidized bed hydrodynamics is necessary for their effective utilization. 

Fine and ultrafine powders, notwithstanding their high surface areas, are often cohesive in nature and difficult to fluidize, making their hydrodynamics poorly understood. This fact was recognized by Geldart [5], who classified particles in different groups based on their fluidization behavior. For example, group A powders, comprised mainly of 30–100 µm particles, exhibit homogeneous expansion leading to particulate expansion when fluidized. On the other hand, fine and ultrafine group C powders are often cohesive and exhibit poor fluidization behavior owing to strong interparticle forces. Cracks and rat holes develop due to the bypassing of fluidizing gas through the beds of these powders, resulting in poor contacting and inefficient interphase mixing [6,7,8]. Although group C particles possess high surface area, their applications in processing using gas–solid fluidization remains challenging because of interparticle forces that render their hydrodynamics unpredictable. This issue has attracted significant attention in the literature. The common strategy to improve the fluidization quality of such powders is mainly to augment additional energy inputs to solids during fluidization to overcome interparticle forces. This strategy enables the elimination of bed nonhomogeneities and reduces powder cohesiveness, thereby improving the quality of fluidization. Strategies for improving fluidization hydrodynamics of powders are often known as assisted fluidization techniques. Examples are acoustics [9,10,11,12,13,14], particle-mixing [15,16,17,18,19,20], flow pulsations [21,22,23,24,25,26,27,28,29], and mechanical vibrations [30,31,32,33,34]. In some cases, a combination of two assisted fluidization technique has been suggested [20,35]. 

Although the efficacy of assisted fluidization techniques is widely reported in laboratory scale setups, its successful implementation for large-scale applications nonetheless depends on its cost effectiveness. Flow pulsation, which involves the pulsating of inlet flow to a fluidized bed at regular time intervals, shows great promise as compared with most other assisted fluidization techniques because additional energy input is not required in its implementation. The bed is subjected to intense periodic turbulence without requiring any additional energy input because of the change in flow rate caused by pulsation. Consequently, it has been gaining significant attention in literature [21,22,23]. When used to dry pharmaceutical granules, this technique decreases the total time of drying while enhancing bed homogeneity as compared with unassisted fluidization [24]. Subjecting a bed of Aerosil 200 nanopowder to flow pulsations substantially lowered the minimum fluidization velocity (*U*_mf_) and improved the bed homogeneity [25]. Another study reported 72% reduction in *U*_mf_ by using pulsed flow [26]. Ali et al. [27] carefully examined the bed collapse behavior and reported a significant decrease in the mean agglomerate diameter that was evaluated from the global pressure transients of the fluidized. Although the fluidization velocity at which the bed collapse was initiated influenced the mean agglomerate diameter, the pulsation frequency on the other hand did not appear to affect its value [28]. The flow pulsation contributed to the reduction of *U*_mf_ and enhancement of the fluidization hydrodynamics of silica powders of different groups of Geldart’s classification [29].

In spite of the widely reported advantages associated with flow pulsation, it has not been extensively investigated. Therefore, the hydrodynamics of the fluidized bed of a hydrophilic ultrafine nanopowder subjected to low-frequency flow pulsation was comprehensively investigated in the present work. Towards this end, we first carefully examined the hydrodynamics of the conventional fluidized bed. No fluidization assistance was applied at this stage of work. Next, we employed flow pulsation by pulsing the inlet flow to the fluidized bed using a square wave of frequency of 0.025-Hz. This strategy involves allowing flow through the fluidized bed for 10 s at various preset velocities followed by 10 s of complete flow interruption, thus introducing periodic sudden expansion and the complete collapse of the fluidized bed. To get a complete picture of the bed hydrodynamics, we monitored bed dynamics in different regions of the fluidized bed using several rapid response sensitive pressure transducers located along the height of the fluidized bed. The experimental system was completely automated. The inlet flow of the fluidizing air was careful controlled using two electronic mass flow controllers that were connected to an 18-bit data acquisition system (DAQ). The voltage signals of the pressure transducers were also monitored using the DAQ. The data sampling frequency was kept at 100 Hz, thus recording 100 data points per second. Given that bed non-homogeneities invariably lead to hysteresis, the fluidization cycles were immediately followed by defluidization by gradually decreasing the flow. We ensured that all the different fluidization stages including the fixed bed mode were covered in our studies starting from zero-flow conditions. The influence of pulsation on fluidized bed hydrodynamics was also explored through the rigorous analyses of bed dynamics in the frequency domain.

## 2. Experimental

**Figure 1:**
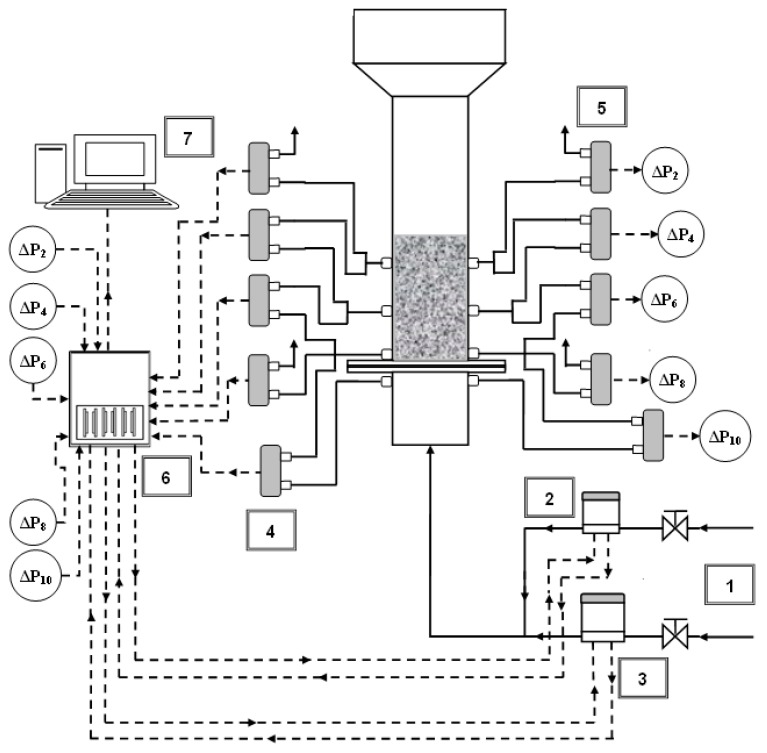
Schematic diagram of the experimental set-up. (**1**) Air supply; (**2**) low range mass flow controller; (**3**) high range mass flow controller; (**4**) pressure transducers (Δ*P*_1_, Δ*P*_3_, Δ*P*_5_, Δ*P*_7_, and Δ*P*_9_ on left side of the column); (**5**) pressure transducers (Δ*P*_2_, Δ*P*_4_, Δ*P*_6_, Δ*P*_8_, and Δ*P*_10_ on the right side of the column); (**6**) data acquisition system; (**7**) computer with LabVIEW software.

Figure 1 shows the schematic of our laboratory-scale experimental set up. A transparent 1.5 m long Plexiglas column with 70 mm internal diameter (ID) was used as the test section. A carefully designed perforated plate distributor with 4% fractional open area was employed to ensure sufficient pressure drop across the distributor plate and eliminate dead zones in the distributor region [36]. Preceding the distributor was a 0.5 m long calming section, while another 0.5 m long disengagement section of 140 mm ID at the top of the column was used to suppress elutriation of particles from the top of the bed. Fluctuation-free high pressure ambient air from a central compressed air supply was used for the fluidization of the nanopowder.

Given that pressure transients reveal crucial information on bed dynamics, its precise and accurate monitoring was ensured by using ten highly sensitive rapid response differential pressure transducers with 1 ms response times. Figure 1 shows that their positions were 11, 110, or 230 mm above the distributor. Two pressure taps located on the diametrically opposite sides of the bed at the same height monitored radial nonuniformities that can arise in the bed. Thus, Δ*P*1 (left side) and Δ*P*2 (right side) monitored the bed dynamics in the upper region of the fluidized bed. Similarly, Δ*P*3 and Δ*P*4 measured the local transients in the central bed regions, whereas Δ*P*5 and Δ*P*6 measured local transients in the lower bed regions. Global bed dynamics was recorded using Δ*P*7 and Δ*P*8. The pressure tap openings were covered with nylon mesh filters to prevent the entrainment of nanoparticles. All the pressure transducers were carefully calibrated using a Fluke 718 pressure calibrator and Fluke voltmeter (Fluke Corporation, Everett, WA, USA).

Two different electronic mass flow controllers were used to monitor and control the flow of the compressed fluidizing air through the test section of the column. We used a 0–20 LPM flow controller for small flow velocities and 0–50 LPM range for controlling high flows. Separate input air supplies were used for each flow controller. Flow controllers preceded the column inlet and were connected to the DAQ. Flow pulsations of 0.05 Hz were generated by controlling the inlet air flow to the test section. National Instruments LabVIEW software (National Instruments, Austin, Texas, USA) was utilized to generate pulsation signals that were transmitted to the DAQ and subsequently to the mass flow controllers as analog outputs in volts. The pressure transducers and flow controllers produced voltage signals, which were recorded at 100 Hz, that is, 100 samples per second, with analog input channels of 18 bit resolution DAQ of National Instruments (USB-6289).

**Figure 2:**
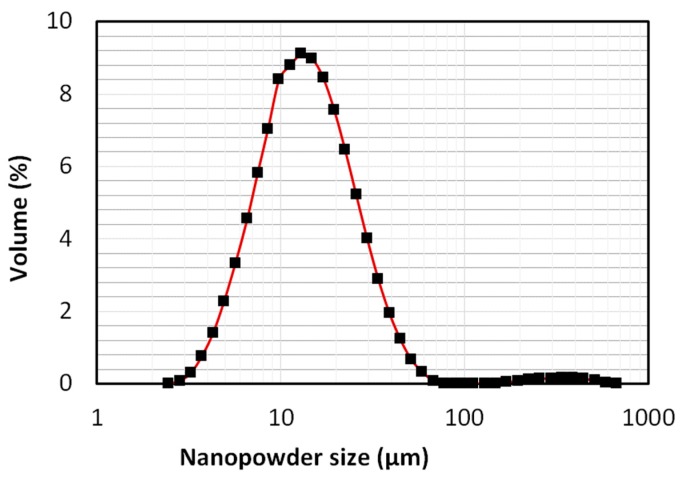
Particle size distribution of the sieved sample of the hydrophilic nanopowder.

**Figure 3:**
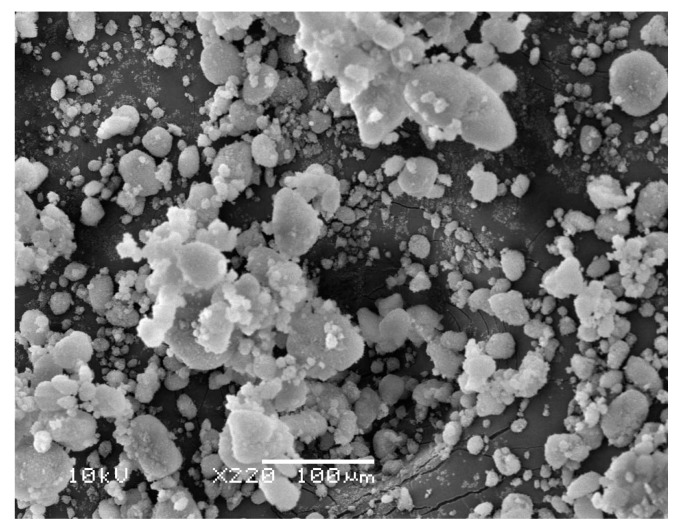
SEM image showing different levels of agglomeration of the nanopowder.

We used 12-nm Aerosil 200 hydrophilic nanosilica (Evonik Industries, Wolfgang, Germany) with a specific surface area of 200 m^2^/g and true density of 2200 kg/m^3^. Fumed nanosilica are widely used in the manufacture of fire protection glass, high-strength concrete, and high-surface area catalysts. When used as rheological additive, it can act as anti-sedimentation and dispersing agent while thixotropizing and thickening the gel or the fluid. This has led to its large scale use in agro-chemicals, battery gels, drilling fluids, sealant, paints and coatings, adhesives, lubricants, and pharmaceutical products. Size analysis and morphological characterization were performed after the sample was carefully sieved for the removal of large agglomerates. A Mastersizer 2000 particle size analyzer (Malvern Panalytical Ltd, Malvern, UK) was employed for dry particle size analysis. The results are presented in Figure 2. A substantial spread in particle size distribution was observed at different levels of agglomeration. The Sauter mean diameter of the present sieved sample was 11.6 µm. Morphological characterization was performed using scanning electron microscope (SEM). Images are shown in Figure 3. The morphology confirms a wide size distribution, as noted in the particle size analysis results.

The flow controllers and pressure transducers were carefully calibrated before the experiments. The column was thoroughly rinsed with an antistatic fluid to minimize the effect of electrostatic on the column walls. The vertical alignment of the column was ensured using a level meter. The samples were sieved to eliminate large agglomerates, which often form during their storage over time. Then, 62 g of the nanopowder (hydrophilic fumed silica) was loaded, and the column wall was frequently tapped. The mixture was left overnight to settle. The static bed height was 370 mm.

**Figure 4:**
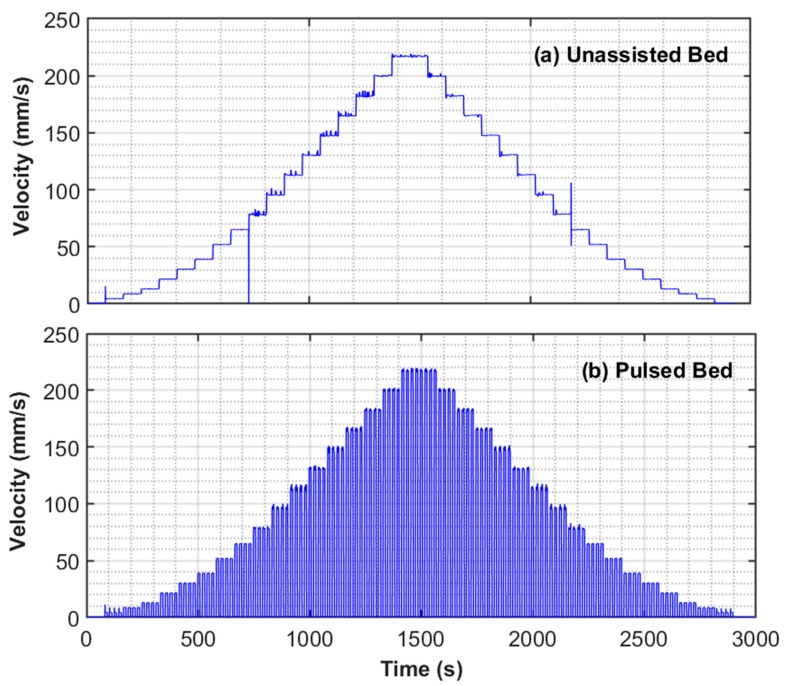
Variation of superficial velocity of the fluidizing air during (**a**) unassisted and (**b**) pulsed assisted fluidization.

**Figure 5:**
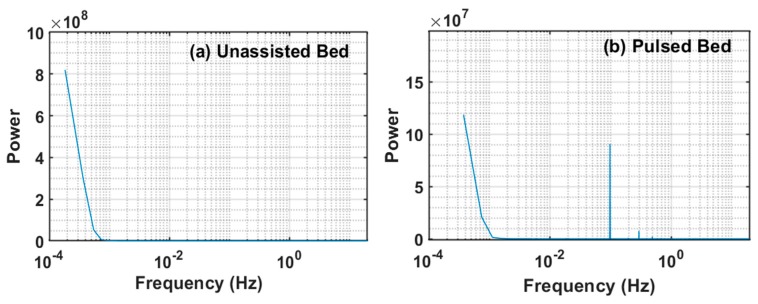
Power spectra of velocity during (**a**) unassisted and (**b**) pulsed assisted fluidization.

Each experiment was composed of a complete cycle that involved gradually increasing (fluidization step) and gradually decreasing velocity (defluidization step), as shown in Figure 4 for both unassisted and pulsed fluidization. The superficial velocity was computed by dividing the flowrate by the cross-sectional area of the test-section of the column. The fluidization cycle of the experiment consisted of 18 stepwise increase in velocity lasting a total of almost 1440 s, followed by a similar stepwise defluidization cycle, as seen in the figure. At every step, whether fluidization or defluidization, the velocity was held constant for 80 seconds. This protocol allowed four 20 s pulses of 0.050 Hz each in the case of pulsed fluidized bed. Each pulse involved 10 s of flow followed by 10 s of flow interruption, as seen in Figure 4. The power spectra of unassisted and pulsed beds are shown in Figure 5. The power spectra peak in unassisted fluidization was almost fourfold of the corresponding value in pulsed fluidization. This result was caused by the regular flow interruptions, reducing the mean velocity to half over the entire pulse duration. The peak seen in the pulsed bed occurred at approximately 0.05 Hz due to flow pulsations. Our experimental strategy of fluidization cycle followed by defluidization helped to delineate the hysteresis effects, thereby giving an improved understanding of the bed homogeneity at different conditions.

**Figure 6:**
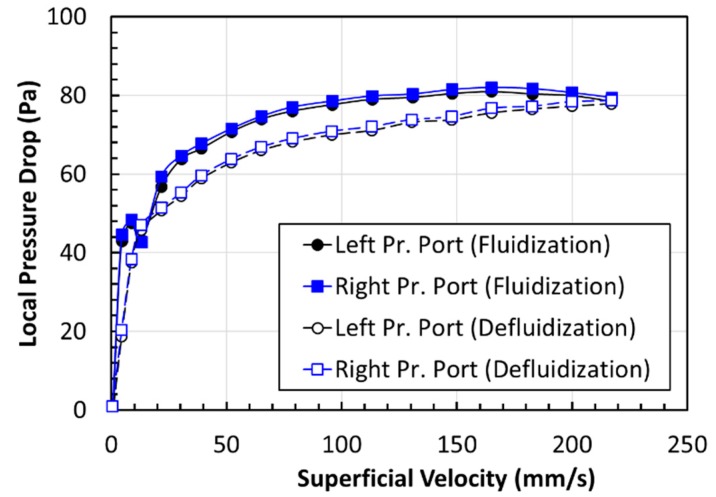
Comparison of pressure drops recorded at the left and right ports in the upper region of the fluidized bed during fluidization and defluidization for unassisted fluidization.

**Figure 7:**
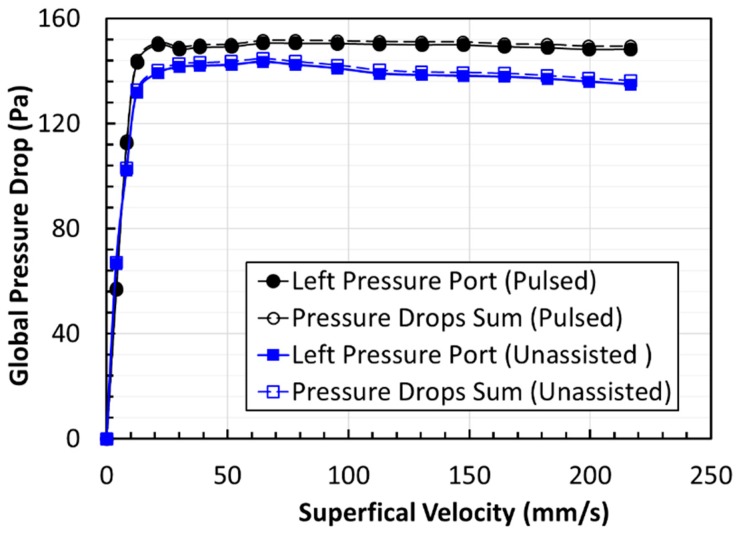
Comparison of the sum of the local pressure drops in the different region of the fluidized bed with the overall pressure drop for both unassisted and pulsed fluidization.

The elutriation of solids from the bed at high velocities sets the upper limit on the air superficial velocity. Two experimental runs were always undertaken to ensure the reproducibility of the experimental data at the same set of conditions. An excellent agreement was mostly observed between runs. Moreover, the fluidized bed was found to be free from radial nonuniformities, as indicated by the excellent agreement seen between the right and left pressure ports in Figure 6. The accuracy pressure drop measurements were further verified by summing local pressure drops in the upper, central, and the lower region of the bed, Δ*P*_1_, Δ*P*_2_, and Δ*P*_3_, respectively, and comparing it with the global or overall pressure drop, that is, Δ*P*_7_, for both cases of assisted and unassisted fluidized beds due to its critical importance in the present experimental investigation. The results of the comparison are depicted in Figure 7. An excellent agreement is seen, confirming the reliability of our measurements of the bed dynamics.

## 3. Results and Discussion

### 3.1. Bed Transients

**Figure 8:**
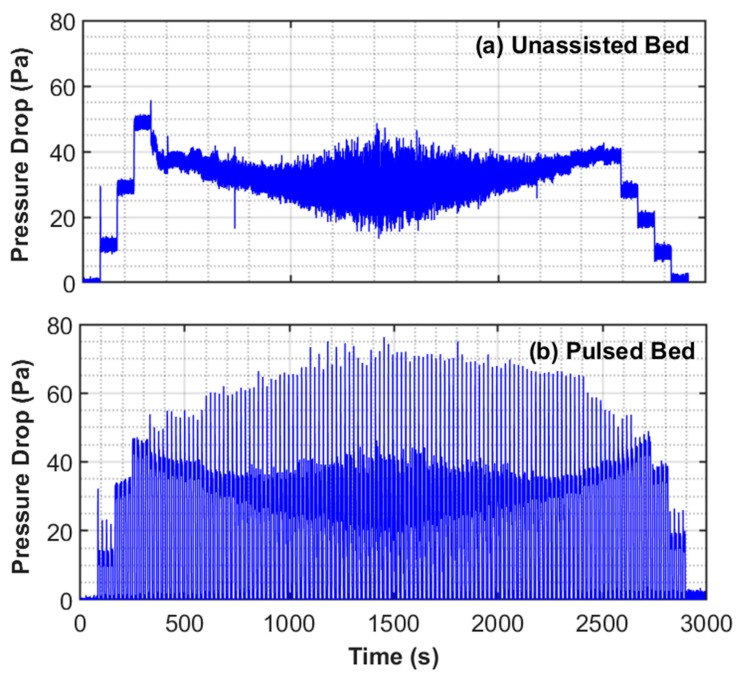
Bed dynamics in the central region of the bed recorded by Δ*P*_3_ for (**a**) unassisted and (**b**) pulsed fluidization.

The bed dynamics of the central region for the entire duration of the experiment in unassisted and pulsed fluidization are presented in Figure 8. An increase in velocity led to a corresponding increase in pressure drop, as clearly indicated in the case of the unassisted fluidization. Its value initially reached as high as 50 Pa before decreasing as the velocity increased. This behavior indicates the onset of fluidization or incipient fluidization. Further increase in velocity after the full fluidization of the bed caused a decrease in the pressure drop from 500 s to 1000 s. This phenomenon was caused by the upward migration of some solids that were initially present in the central region of the fluidized bed due to the expansion of the bed. The pressure drop remained relatively constant at high velocities from 1000 s to 1400 s because the upward migration of solids from the central region was replenished by the input of the mass from the lower regions of the bed, which was fluidized last because of the presence of large agglomerates.

Another feature of the unassisted fluidization is the occurrence of high frequency fluctuations, which tend to increase with velocity in the later stages of the fluidization cycle and early stages of the defluidization cycle. The reverse flow of solids was observed from the upper regions owing to a decrease in bed expansion as velocity decreased during the defluidization from 2000 s to 2600 s. At corresponding velocities, smaller pressure drops were noted as compared with ones seen during the fluidization, with further decreases in velocity after 2600 s. This result can be attributed to removal of nonhomogeneities from the bed that led to a smoother defluidization process.

In the pulsed-fluidized bed, the transients were dominated by the response of the bed to flow interruptions. The sudden flow of fluid through the bed interstices led to a spike in the pressure drop when the flow began after a pause of 10 s at the preset velocity. The mean of this steady value was found comparable with the one obtained for the unassisted fluidization at corresponding flowrates.

### 3.2. Pulsed Bed Transients

**Figure 9:**
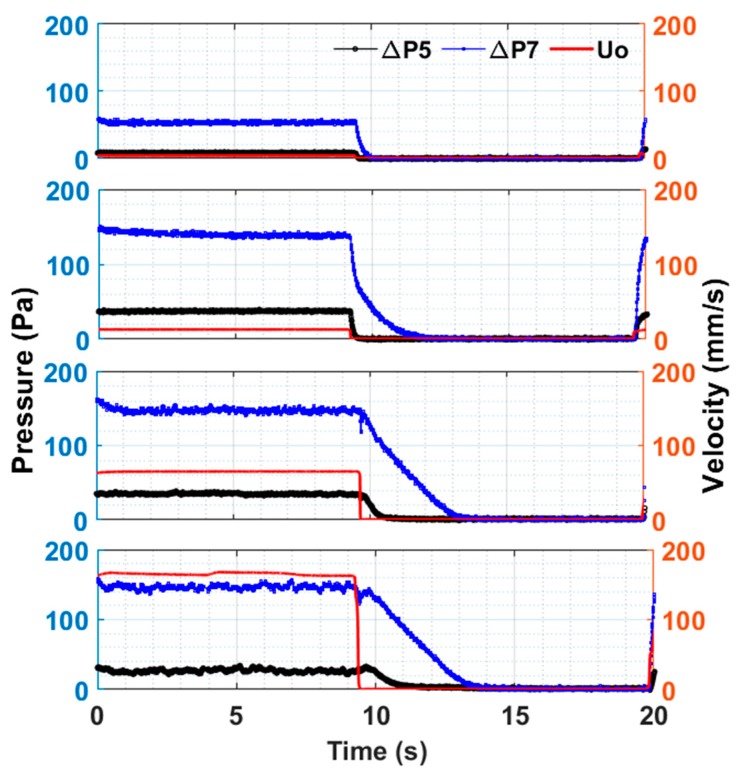
Global ($\Delta P_{7}$ and local (lower region, $\Delta P_{5}$) bed dynamics in pulsed fluidized bed for 4 mm/s, 12 mm/s, 65 mm/s, 165 mm/s during fluidization.

We considered only one pulse in Figure 9 to have a closer look on the pulsed bed dynamics, which showed the pressure transients in the lower region of the fluidized bed in addition to the overall pressure drop transients at four different velocities. The right-hand side of the scale depicts the velocity profile in mm/s. The solid–fluid contacting was in the fixed bed mode at 4 mm/s. The pressure drop was low because the velocity was low. A threefold increase in velocity to 12 mm/s led to a several fold increase in pressure drop in Δ*P*_5_ and Δ*P*_7_ because the pressure drop, as described by the Ergun equation, was linearly dependent on the velocity in the laminar flow regime. It showed strong dependence on velocity in the laminar flow regime. However, another fivefold increase in the velocity to 65 mm/s led to a relatively insignificant rise in pressure drop because of the onset of fluidization. The pressure drop remained constant and was equal to the effective bed weight when the velocity increased further to 165 mm/s. However, fluctuations in the pressure transients were clearly visible, unlike those at lower velocities. These fluctuations were introduced due to the vigorous motion of solids at high velocities.

When the flow to the fluidized bed was cut off, the bed immediately started to fall with a decrease in the pressure drop. Although bed collapse was initiated at different velocities (e.g., 65 and 165 mm/s), the time of the fall of the bed remained nearly same despite several fold differences among velocities. The bed required approximately 5 s for complete settling in the present case. This result was, however, significantly shorter for the lower region of the fluidized bed.

**Figure 10:**
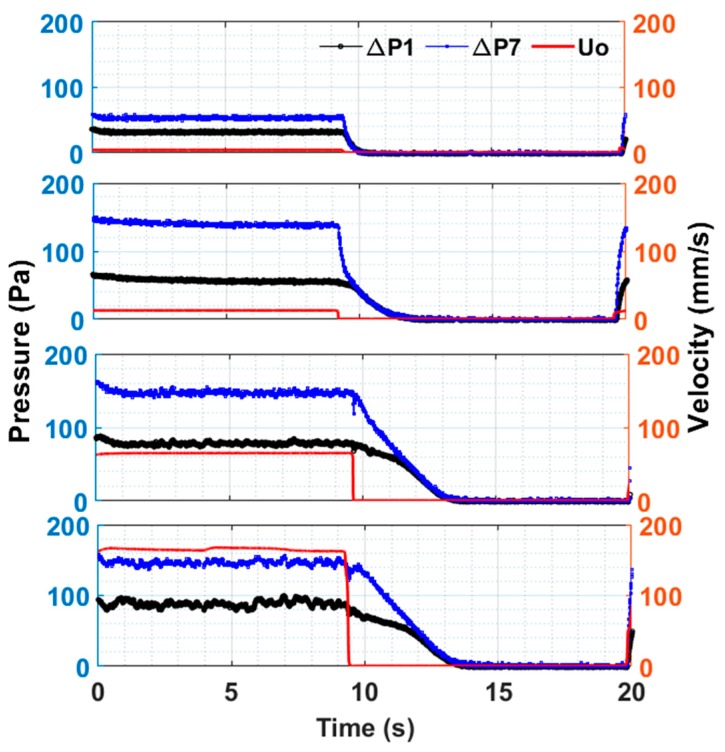
Global ($\Delta P_{7}$ and local (upper region, $\Delta P_{1}$) bed dynamics in pulsed fluidized bed for 4 mm/s, 12 mm/s, 65 mm/s, 165 mm/s during fluidization.

The bed dynamics of the upper region of the fluidized bed and its overall dynamics are shown in Figure 10. Despite obvious similarities with Figure 9, an important difference was observed between the hydrodynamics of the two regions. The upper region showed slower transients than the lower regions of the fluidized bed. The two pressure transients, that is, upper and global, merged after approximately 2.5 s since the start of the bed collapse. This finding indicates that the collapse process was controlled by the upper region of the bed.

### 3.3. Mean Pressure Drop

**Figure 11:**
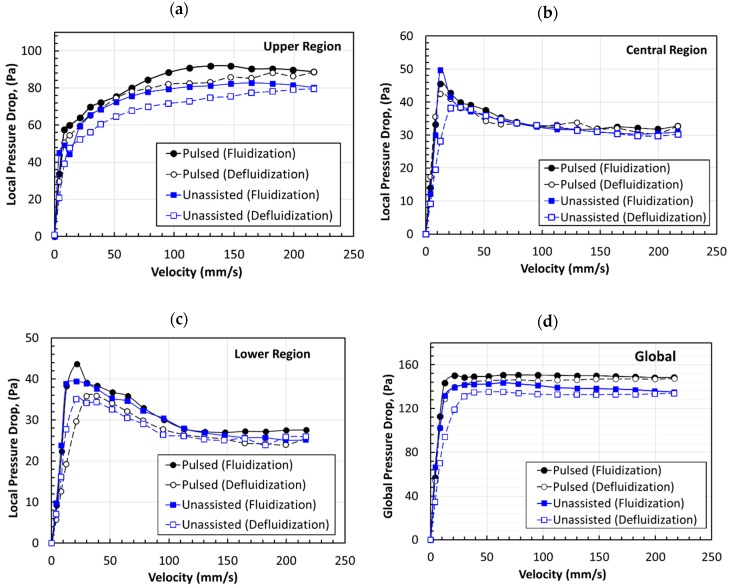
Dependence of the pressure drop on the velocity for different regions of the unassisted and pulsed fluidized beds during fluidization and defluidization, (**a**) upper region, (**b**) central region, (**c**) lower region, (**d**) overall bed.

Figure 11 shows the variations in local pressure drop and velocity for all the three different regions and the overall fluidized bed. The results for fluidization and defluidization cycles are presented together for the analysis of the effect of the hysteresis phenomenon. The upper region of the fluidized bed showed a progressive increase in pressure drop with the increase in velocity in the unassisted and pulsed-fluidized beds. This phenomenon was caused by the upward movement of the mass from the lower regions into the upper one as a consequence of the bed expansion. Given that the fluidized bed pressure drop was an effective weight of the solids present between the pressure ports, an increase in solid mass in the upper region was reflected in the increase in the pressure drop. Another notable feature of Figure 11a is the higher pressure drop in the pulsed bed as compared with that in the unassisted bed. This result indicated a large bed expansion during pulsed fluidization. Moreover, the pulsed fluidization tended to mitigate the hysteresis phenomenon.

The central region of the fluidized bed showed pronounced hysteresis initially during unassisted fluidization with as much as 70% difference in the pressure drop between the fluidization and the defluidization when the velocity was 12 mm/s (Figure 11b). On the other hand, flow pulsation substantially reduced the hysteresis.

The lower region of the fluidized bed showed good agreement between the unassisted and pulsed fluidized beds during fluidization and defluidization (Figure 11c). In this case, however, unlike the previous two cases, the hysteresis appeared more pronounced for the pulsed bed, especially at low velocities. Nevertheless, attributing the present hysteresis-like phenomenon to bed nonhomogeneities is misleading. This phenomenon was due to the migration of mass from the lower region of the bed to the upper regions because of the deagglomeration of large agglomerates present in this part of the pulsed fluidized bed, thereby leading to large bed expansion.

Figure 11d shows the comparison of the overall pressure drop across the entire bed height for unassisted and pulsed fluidized beds. Clearly, the flow pulsation substantially suppressed the hysteresis. Moreover, the pressure drop was higher for the pulsed fluidization, which was a clear indication of elimination of bed non-homogeneities that caused local by-passing of air. The pressure drop for the pulsed bed during the defluidization cycle was higher at low velocities than corresponding values in its unassisted counterpart in the fixed bed mode of contacting. This result is possible only if the particles or the agglomerates are small. Thus, the flow pulsation promoted deagglomeration. This aspect is discussed later in Section 3.4.

### 3.4. Minimum Fluidization Velocity

**Figure 12:**
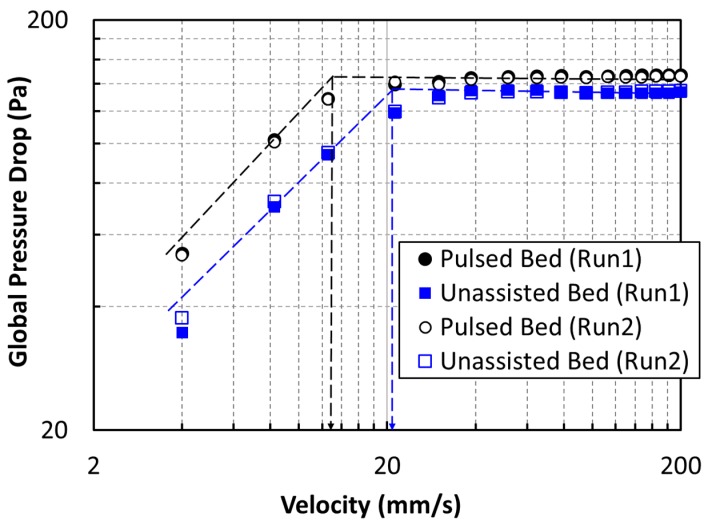
Global pressure drop variation with velocity for the defluidization cycle of experiments for determining *U*_mf_.

Figure 12 presents the global pressure drop profiles of defluidization cycles of both experimental runs for the determination of the minimum fluidization velocity, *U*_mf_. Although the excellent agreement between two runs highlights the reliability of our experimental data, the unassisted fluidization yielded *U*_mf_ = 22 mm/s. Meanwhile, the value obtained using pulsed fluidization was 14 mm/s. This difference was more than 50%. The lowering of the *U*_mf_ is a clear indication of deagglomeration due to flow pulsation. Previously reported *U*_mf_ values were 118.3 and 30.5 mm/s for unassisted fluidization and 10-s pulsation, respectively [22]. This difference can be attributed to the removal of large agglomerates due to sieving of the nanopowder sample in the present experiments.

### 3.5. Mean Effective Agglomerate Diameter

**Figure 13:**
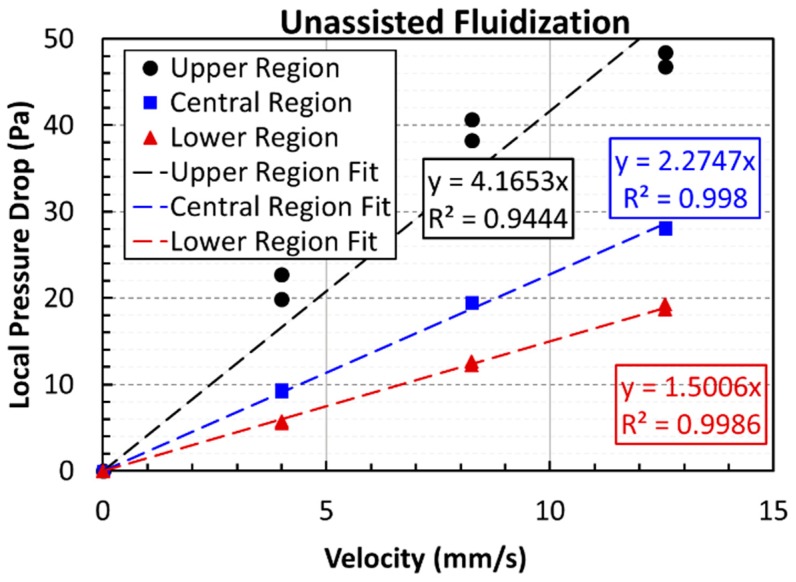
Local pressure drop variations with velocity and corresponding linear-fits for the defluidization cycle of unassisted fluidized bed.

The effect of pulsation on the deagglomeration was evaluated by computing the mean effective diameter from the pressure drop data during the defluidization cycle of the experiment. The well-established Ergun equation that describes the dependence of the pressure drop on the fluid velocity and particle size in the fixed bed mode of contacting was employed. Results are presented in Figure 13 for all the three different regions of the unassisted fluidized bed. Data from both experimental runs were used and fitted with straight lines. The values of the slopes and their coefficients of determination, *R*^2^, are also shown in the figure. The slope of the pressure drop versus velocity profile was higher in the upper region than that the slopes in the central and lower regions of the fluidized bed. The fitted slope values were utilized for the evaluation of the mean effective diameter through the following Ergun equation-based relationship:

(1) $$\frac{\Delta P}{U_{0}}=\frac{150\mu }{D_{Pm}^{2}}\frac{{\left(1-\varepsilon \right)}^{2}}{\varepsilon^{3}}\Delta L$$

where Δ*P* (Pa) is the pressure drop across the bed length Δ*L* (m) at the fluid superficial velocity of *U*_0_ (m/s) for the bed void fraction (*ε*) and fluid viscosity, *µ* (Pa·s) when the mean effective agglomerate diameter is *D_Pm_* (m). The bed void fraction was computed here from the bed height, solid density, and the total mass of the nanopowder present in the bed. Our experimental data yielded 7.2, 9.0, and 10.1 µm as the mean effective agglomerate diameter for the upper, central, and lower regions, respectively. Thus, a clear evidence of segregation based on the size of the agglomerates was observed. The average size of the nanoagglomerates in the upper region was more than 40% smaller than that in the lower region. The agglomerates in the central region were 10% smaller than those in the lower region. The occurrence of size segregation in the nanopowder bed was reported previously and was observed through visual observations [6]. The extent of size-segregation is now clear with our reported results.

**Figure 14:**
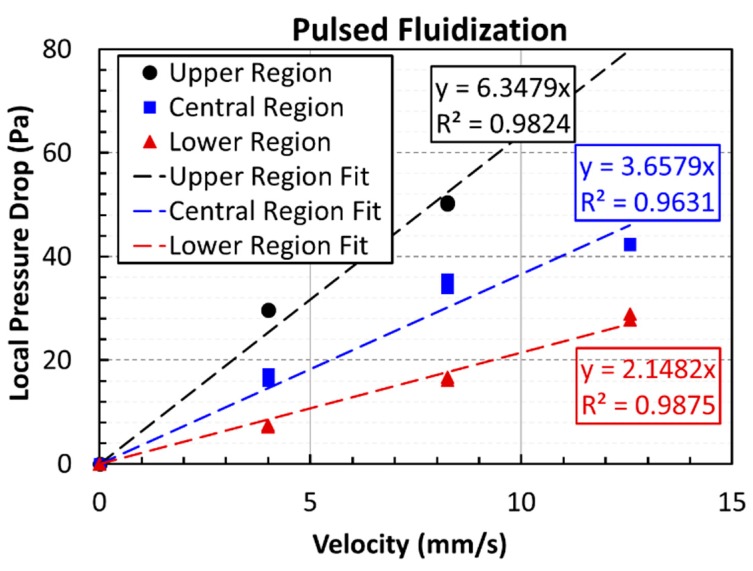
Local pressure drop variations with velocity and corresponding linear-fits for defluidization cycles of pulsed fluidized bed.

Figure 14 shows the data of defluidization cycles of both experimental runs for the pulsed fluidized bed. The trend of the pressure drop in the different regions of the pulsed fluidized bed was similar to the previous case of the unassisted fluidized bed. The slopes and the *R*^2^ values are also shown in the figure. The mean effective agglomerate diameters were 5.8, 7.1, and 8.4 µm for the upper, central, and lower regions, respectively. The decrease in the diameter, being 43% and 16% for the central and upper regions, respectively, was a little higher than that in the unassisted case when compared with the size of the nanoagglomerates present in the lower region of the pulsed bed.

**Figure 15:**
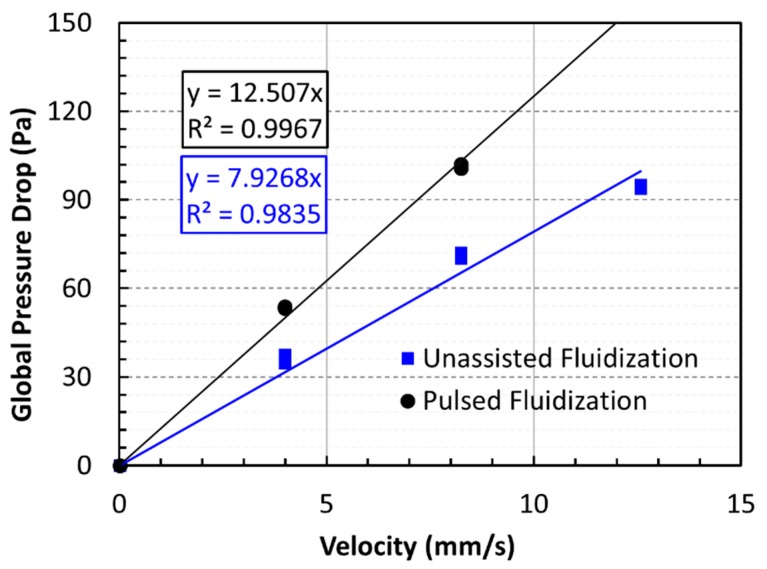
Global pressure drop variations with velocity and corresponding linear-fits for the defluidization cycles of unassisted and pulsed fluidized beds.

Figure 15 compares the global pressure drops for unassisted and pulsed fluidization. Data of both experimental runs were fitted with straight lines like before. *R*^2^ values indicate good agreement, shown in the figure. Higher slope was obtained for the pulsed fluidization, which was almost 90% higher than the unassisted fluidization. Agglomerates sizes were 6.6 µm and 8.4 µm for the pulsed and unassisted fluidization, respectively. The difference between the two was more than 20%. The results are summarized in Table 1. The last column of the table shows the percent reduction in the agglomerate size after the mean effective diameter was compared with the average size obtained from the particle size analysis. While unassisted fluidization caused size reduction as high as 28%, the flow pulsation promoted higher deagglomeration with almost 43% size reduction when the global pressure drop data was used in computing the mean effective diameter.

### 3.6. Comparison of Frequency Response

**Figure 16:**
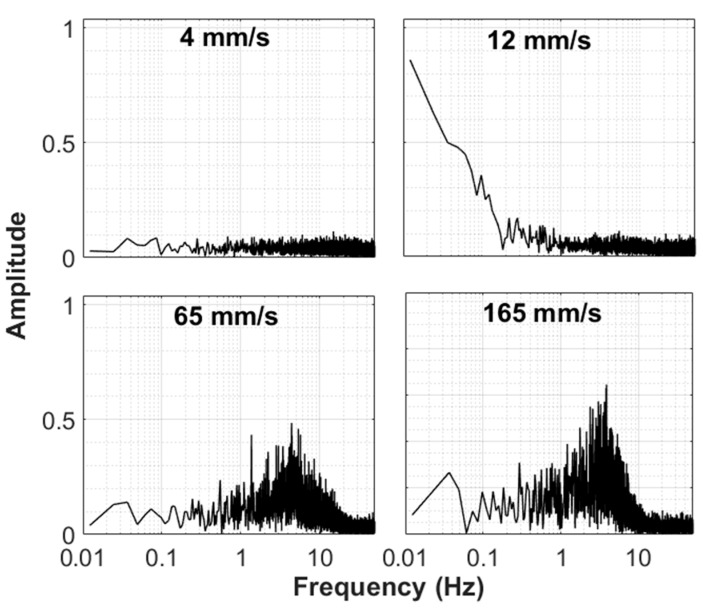
Frequency response of overall bed dynamics at different velocities of fluidization cycle for unassisted fluidization.

The frequency response of the fluidized bed at different velocities for the case of the unassisted fluidization is shown in Figure 16. The detrended global bed dynamics data were employed in the present study. The term detrending means removal of any trend from the data. The mean value of the data series considered for frequency analysis was zero. The bed was stationary at a low velocity (4 mm/s), so the amplitudes of disturbances were almost negligible. The overall bed was still stationary (fixed bed state) at 12 mm/s. Nevertheless, a prominent relatively large amplitude low frequency peak was observed because of the eruption of intermittent bubbles at the top of the surface of the bed after a long pause between successive eruptions. This conclusion was formed after examining the frequency response of all the three different regions of the fluidized bed (Figure 18). A similar peak was observed only in the upper region of the fluidized bed and not in the central and lower regions of the fluidized bed. Disturbances were observed, which mainly occurred in a frequency range of 1–10 Hz at 65 mm/s when the bed of hydrophilic nanosilica was fully fluidized. These disturbances were due to agglomerate motion caused by the passage of the air through the bed. When the velocity was increased to 165 mm/s, vigorous motion in the bed agglomerates was observed and indicated by high amplitude fluctuations.

**Figure 17:**
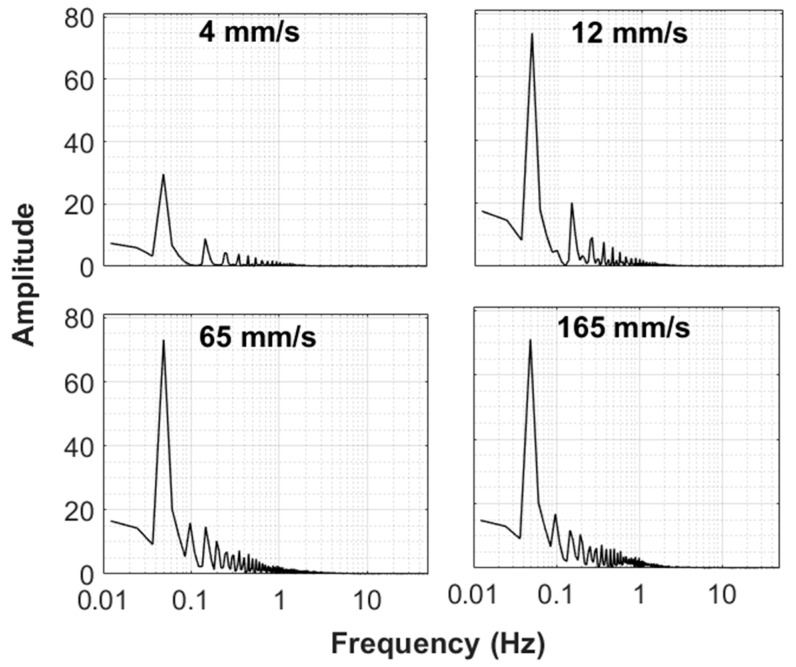
Frequency response of overall bed dynamics at different velocities of fluidization cycle for pulsed fluidization.

The frequency response of the pulsed-fluidized bed was examined using the detrended data in Figure 17. Instead of local transients, our attention was mainly focused on the overall bed dynamics in this study because localized events can be represented by global bed dynamics, as seen earlier for the case of bubble eruption at the top surface of the bed. The bed dynamics were now essentially dominated by the pulsation dynamics. The most prominent peak was due to the flow pulsation introduced in the bed. Many smaller peaks, visible up to 1 Hz, were the after-effects of the pulsation. The peak at 4 mm/s was smaller than the corresponding peaks at higher velocities due to lowered pressure drop. Despite the difference in velocities, the magnitude of the amplitude remained unaffected because the pressure drops were hardly affected by the velocity change after 12 mm/s during pulsed fluidization, as seen in Figure 11d. Nonetheless, the pulsation after-effects were apparently mitigated by the vigorous solid motion prevailing at high velocities.

**Figure 18:**
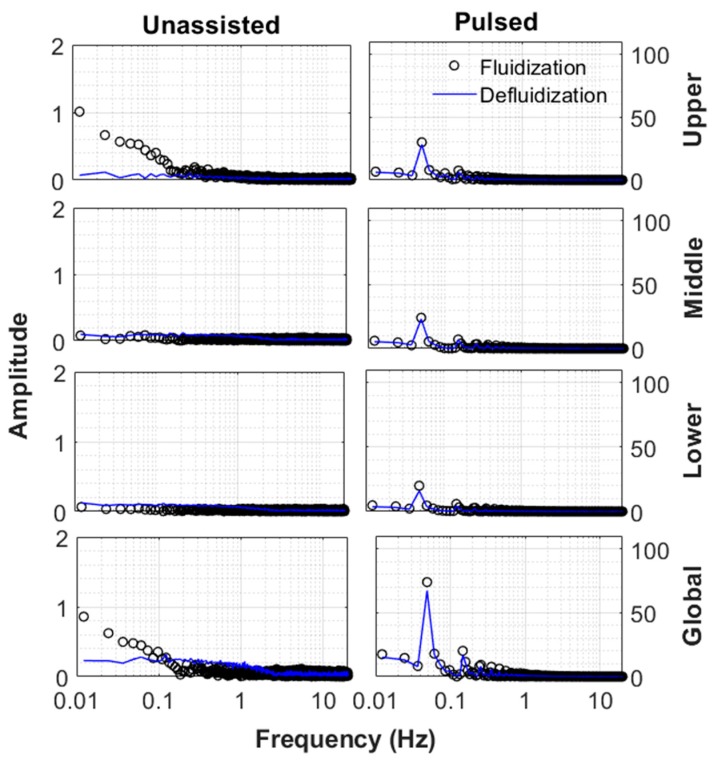
Frequency response of different regions of the bed at 12 mm/s for both unassisted and fluidization.

**Figure 19:**
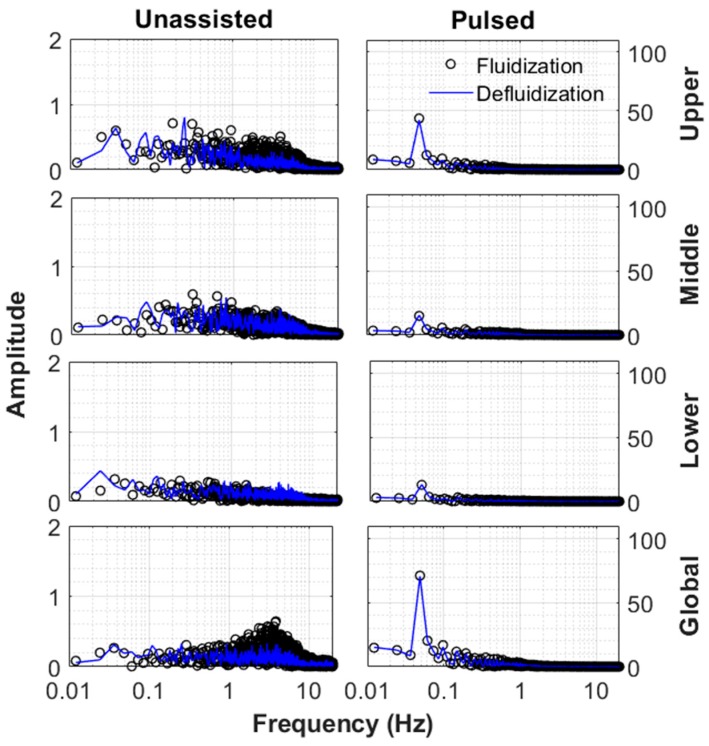
Frequency response of different regions of the bed at 165 mm/s for both unassisted and fluidization.

The difference between the dynamics of the fluidization and defluidization cycles were examined for unassisted and pulsed fluidizations. Results are depicted in Figure 18 for 12 mm/s, which was slightly below the minimum fluidization velocity. On the other hand, Figure 19 shows the case of 165 mm/s when the bed was fully fluidized. In the upper region of the unassisted fluidized bed, a difference between the fluidization and defluidization cycles was observed at low velocities. Although the bed did not appear to be fluidized, the agglomerates present in the upper region of the bed, being small, were disturbed due to the drag of the fluidizing air. This effect was nonetheless significantly mitigated during the defluidization cycle because the agglomerates tended to rearrange themselves to lower the friction with the air flow. There was an interesting occurrence of low frequency phenomenon with relatively higher amplitude in the upper region of the unassisted fluidized bed at 12 mm/s, which was not seen in the middle and lower sections of the fluidized bed. This phenomenon can be attributed to the intermittent bubble eruption at the top of the bed. On the contrary, a surprising agreement was observed between the fluidization and defluidization cycles of the pulsed fluidization as regular flow interruptions caused the agglomerates to rearrange themselves and thereby removed nonhomogeneities. This phenomenon appeared to be more pronounced at 165 mm/s. Figure 19 shows that the motion of agglomerates tended to decrease as the distance from the distributor decreased during the fluidization cycle during unassisted fluidization. Moreover, the defluidization cycle imparted homogeneity as the bed dynamics showed smaller fluctuations. Pulsed fluidization tended to eliminate bed nonhomogeneities, leading to surprising agreement between the fluidization and defluidization cycles.

## 4. Conclusions

The effect of low-frequency flow pulsations on the hydrodynamics of the fluidized bed were rigorously investigated by carefully comparing its behavior with one of the unassisted fluidization for the delineation of the effects after the pulsating of the inlet flow of fluidizing air. The term “low frequency” indicates the sufficiently long pause between successive pulsations to fully settle and reach equilibrium. The bed transients in the different regions of the fluidized bed were examined. The bed collapse behavior was found to be controlled by the upper region of the pulsed-fluidized bed. The pulsations improved bed homogeneity and led to almost 40% reduction in the *U*_mf_. Moreover, the strategy of flow pulsations greatly promoted the deagglomeration of nanoagglomerates, leading to as much as 42.7% overall reduction in agglomerate size when compared with the size of the sample obtained from the particle size analysis. The size reduction was as high as 50% in the upper region as compared to 27% in the lower region of the pulsed fluidized bed. This phenomenon can be attributed to the fact that the collapse dynamics during flow interruption was controlled by the upper region.

We also analyzed the frequency response of both unassisted and pulsed fluidized beds. The results revealed interesting insight into the bed dynamics that were otherwise not evident from the real-time pressure transients. The occurrence of events of different frequencies and their amplitudes were clearly delineated. The pulsed bed was dominated by the pulsation event and its high frequency after-effects, which progressively decreased in amplitude at high frequencies. A comparison between fluidization and defluidization cycles of pulsed and unassisted fluidized beds showed that pulsation improved hydrodynamics by imparting greater homogeneity to the fluidized bed.

## Figures and Tables

**Table 1:** Effective agglomerate diameter in different regions of the fluidized bed and percent reduction using particle diameter obtained using particle size analysis.

	Region	Slope	*R* ^2^	Diameter	Reduction
		(Pa/(m/s))		(µm)	(%)
Unassisted fluidization	Upper	4165.3	0.9444	7.2	38.0
	Central	2274.7	0.998	9.0	22.3
	Lower	1500.6	0.9986	10.1	13.1
	Global	7926.8	0.9835	8.4	28.0
Pulsed fluidization	Upper	6347.9	0.9824	5.8	49.8
	Central	3657.9	0.9631	7.1	38.7
	Lower	2148.2	0.9875	8.4	27.4
	Global	12507	0.9967	6.6	42.7
